# Dioxygen insertion into the gold(i)–hydride bond: spin orbit coupling effects in the spotlight for oxidative addition†

**DOI:** 10.1039/c6sc02161a

**Published:** 2016-07-25

**Authors:** Carlo Alberto Gaggioli, Leonardo Belpassi, Francesco Tarantelli, Daniele Zuccaccia, Jeremy N. Harvey, Paola Belanzoni

**Affiliations:** a Department of Chemistry, Biology and Biotechnology, University of Perugia via Elce di Sotto, 8 06123 Perugia Italy paola.belanzoni@unipg.it; b Institute of Molecular Science and Technologies (ISTM) – CNR via Elce di Sotto, 8 06123 Perugia Italy; c Department of Food, Environmental and Animal Sciences, Section of Chemistry, University of Udine via Cotonificio, 108 33100 Udine Italy; d Department of Chemistry, KU Leuven Celestijnenlaan 200F, B-3001 Heverlee Belgium jeremy.harvey@chem.kuleuven.be

## Abstract

O_2_ insertion into a Au(i)–H bond occurs through an oxidative addition/recombination mechanism, showing peculiar differences with respect to Pd(ii)–H, for which O_2_ insertion takes place through a hydrogen abstraction mechanism in the triplet potential energy surface with a pure spin transition state. We demonstrate that the spin-forbidden Au(i)–hydride O_2_ insertion reaction can only be described accurately by inclusion of spin orbit coupling (SOC) effects. We further find that a new mechanism involving two O_2_ molecules is also feasible, and this result, together with the unexpectedly high experimental entropic activation parameter, suggests the possibility that a third species could be involved in the rate determining step of the reaction. Finally, we show that the O_2_ oxidative addition into a Au(i)–alkyl (CH_3_) bond also occurs but the following recombination process using O_2_ is unfeasible and the metastable intermediate Au(iii) species will revert to reactants, thus accounting for the experimental inertness of Au–alkyl complexes toward oxygen, as frequently observed in catalytic applications. We believe that this study can pave the way for further theoretical and experimental investigations in the field of Au(i)/Au(iii) oxidation reactions, including ligand, additive and solvent effects.

## Abstract 2

O_2_ insertion into a Au(i)–H bond occurs through an oxidative addition/recombination mechanism that can only be described accurately by inclusion of spin orbit coupling (SOC) effects.

## Graphical abstract

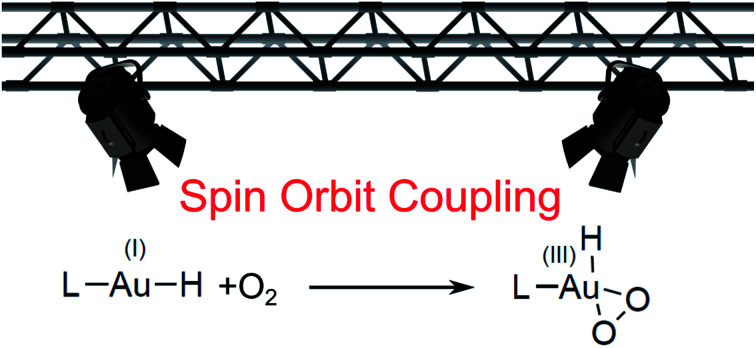

## Introduction

**Fig. 1:**
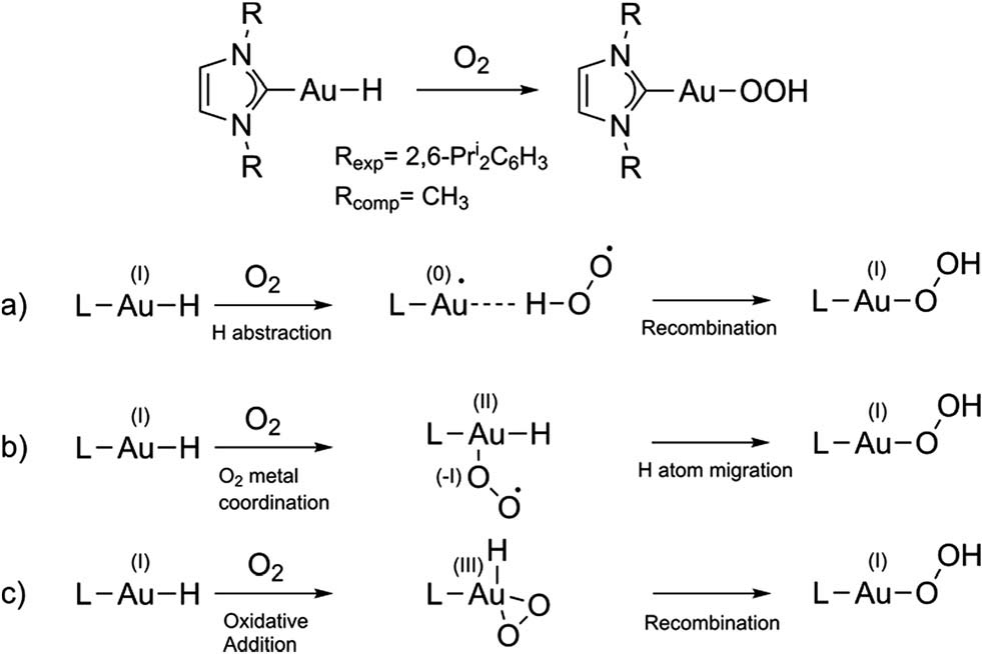
From top to bottom: experimental (and computational model) reaction of gold(i) complex with O_2_ and sketch of the analysed mechanisms. (a) Hydrogen abstraction mechanism; (b) O_2_ metal coordination mechanism; (c) oxidative addition/recombination mechanism.

Controlled activation of molecular oxygen is one of the biggest challenges in catalysis since O_2_ is kinetically quite stable, but it is highly desirable in order to obtain a green, and easily accessible chemical oxidant to displace the currently used expensive, polluting and dangerous oxidants.^1^ Great effort has been devoted to finding catalytic systems aimed at more efficiently activating O_2_ for its use in catalysis, leading in particular to the development of transition-metal catalysts^2^ and aerobic oxidations using palladium complexes.^3^ In the catalytic cycle of the latter reactions, a Pd–hydride bond is formed, which reacts with O_2_ forming a hydroperoxide compound. Although most of the work has focused on Pd (and some on Pt based complexes^4,5^), also some examples using gold compounds for activating O_2_ are known, both in heterogeneous^6,7^ and homogeneous catalysis.^8^ Homogeneous gold-catalyzed reactions in which gold hydride precursors and intermediates are considered key compounds include, for instance, hydrodefluorination of perfluoroarenes,^9^ dehydrogenative alcohol silylation^10^ and enantioselective hydrogenation of alkenes and imines.^11^ All these works attest to the relevance of the metal hydride bond and O_2_ interactions in many important catalytic cycles. The reaction of Au(i) complexes with O_2_ is key for the oxidative addition to gold(i), forming gold(iii), which has been defined by Teles as a new avenue in homogeneous catalysis with Au.^12^ Oxidative addition was until recently considered to be very unfavorable, if not impossible, with gold, while ubiquitous for palladium, because of the high redox potential of the Au(i)/Au(iii) pair compared to that of isoelectronic Pd(0)/Pd(ii). However, recent studies have highlighted other structural and electronic factors that can play a major role in promoting Au(i) to Au(iii) oxidations.^13–16^ In contrast with the many computational mechanistic studies on Pd complexes, to our knowledge no computational studies aimed at understanding the activation of O_2_ using gold-hydride species and at exploring in detail the possibility of O_2_ performing oxidative addition to Au(i) complexes have been undertaken so far. Recently, O_2_ addition to a gold(i) hydride was shown to form a hydroperoxide^17^ (Fig. 1 top).

Here, we theoretically explore the mechanism of this reaction. The overall spin state of the reactants is triplet (triplet O_2_ and singlet Au(i) hydride), while the product has a singlet ground state, so the reaction is spin-forbidden.

## Methodology

**Fig. 2:**
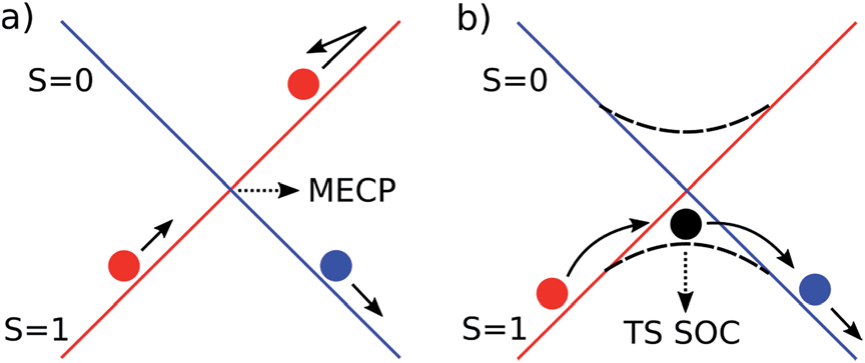
Schematic representation of the two limiting scenarios for a spin-forbidden reaction; (a) diabatic PESs crossing at an MECP; (b) adiabatic PES with changing spin character.

Spin orbit coupling (SOC) effects are expected to be important in this reaction^18^ for species in which unpaired electron density resides on the heavy gold atom. Spin-forbidden reactions^19^ can be described as involving ‘hopping’ from the diabatic potential energy surface (PES) of one pure spin state, to another (Fig. 2a), or as involving smooth transition from one spin state to another along an adiabatic reaction path (Fig. 2b). The first description is more accurate when SOC is weak. Hopping takes place in this case near the minimum energy crossing point (MECP) between the diabatic surfaces. The second description is preferred when SOC is strong, and in this case there can be an adiabatic saddle-point (or transition state TS SOC) near where the transition occurs.

We performed DFT calculations using a simplified model of the gold(i) complex, with the Ar groups on the 1,3-bis(2,6-diisopropylphenyl)-imidazol-2-ylidene ligand replaced by methyl groups. The predictive accuracy of the model has been validated through comparative calculations on the full experimental system (see Fig. S3 in the ESI†). All calculations to locate reactants, transition states, intermediates and products involved in the considered mechanisms have been done with the ADF 2012.01 program package,^20^ using the following details: BP86 functional,^21^ grimme3 BJ damping dispersion effect (DFT-D3-BJ),^22^ a Slater-Type DZP quality (TZP for gold) basis set and a ZORA Hamiltonian^23^ to include relativistic effects. We employed either the scalar or the spin–orbit ZORA Hamiltonian, the latter used in order to take into account spin orbit coupling effects. If not specified, energies refer to scalar ZORA calculations. The computation of the minimum energy crossing points (MECP) has been done using a program developed by one of us^24^ interfaced with the ADF package, with the computational details described above. All calculations have been performed under vacuum. Whenever we calculated Δ*G*, we computed frequencies for the involved reactants, intermediates, transition states and product structures. All computed transition states have only one imaginary frequency. The gas-phase Δ*G* is calculated as the sum of Δ*H* and −T*Δ*S* at 298.15 K, where Δ*H* is the sum of Δ*E* and Δ(ZPE) (Zero Point Energy).

Additional calculations (see the ESI, Fig. S5, Tables S1–S4†) have been performed to substantiate the reliability of the level of theory we used to describe the system for the purpose of this work, assessing the possible error bars one would get by changing any basis set, exchange-correlation functional, D3-BJ dispersion correction or solvation (benzene) item. Qualitatively, we find that the results are not sensitive to the choice of methodology, though the magnitude of the energy barriers changes somewhat, as discussed below.

## Results and discussion

We selected the investigated mechanisms on the basis of what was reported in ref. 3, with the precise aim of comparing similar reaction mechanisms to those reported for O_2_ insertion into Pd(ii) hydride bonds, as well as a novel mechanism involving two O_2_ molecules. We note that in order to explain the reactivity^25^ of Au(i)–H and Au(iii)–H bonds,^26^ concerted^10^ and trimolecular mechanisms were often invoked.^9,11*b*,27^ The alternative of a radical reaction has been discarded on the basis of experiments conducted in the presence of a radical scavenger (TEMPO and galvinoxyl radical) which exclude the reaction of O_2_ with (IPr)AuH proceeding *via* a radical chain mechanism.^17^Fig. 1 (bottom) shows the three investigated mechanisms: hydrogen abstraction by O_2_ to form a radical pair intermediate, which then recombines to form the hydroperoxyde (Fig. 1a); coordination of O_2_ to gold, then insertion into the Au–H bond (Fig. 1b); and oxidative addition of O_2_ to the gold centre to produce a formal gold(iii) intermediate followed by reductive elimination (or recombination, Fig. 1c). We located the MECPs using a program developed by one of us,^24^ interfaced with the ADF package, and the TSs by employing the ZORA SOC Hamiltonian.

**Fig. 3:**
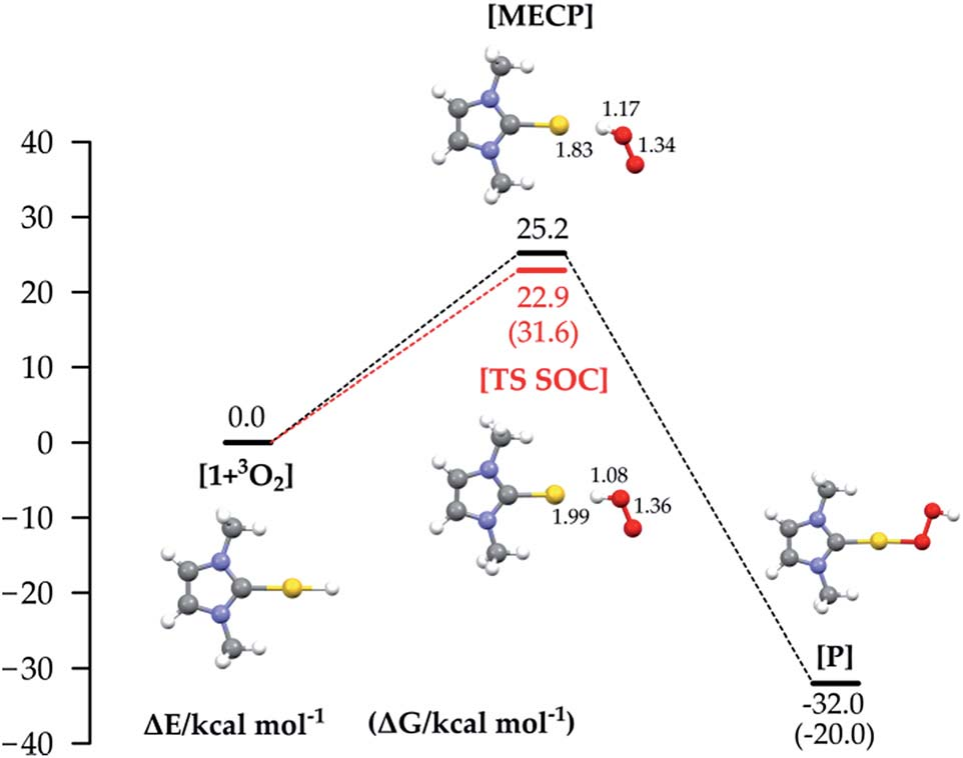
Reaction energy profiles and structures for the hydrogen abstraction mechanism (the red line shows the energy of the TS optimized with SOC). Δ*G* values are shown in brackets. All energy values (in kcal mol^−1^) refer to the energy of the isolated reactants taken as zero.

For mechanism 1a, we first considered scans of the triplet and singlet potential energy surfaces as O_2_ approaches the hydride, which leads to a crossing of the triplet state, lower at large O–H, and the singlet state, which starts out higher but leads ultimately down to the product after hydrogen abstraction with facile rotation of the OOH group (as in Pd analogues^3^) (see Fig. S1 in the ESI†). An associated MECP was then located, as well as an adiabatic TS when using ZORA SOC. The MECP and TS are very similar to one another both in energy and geometry (Fig. 3).

The reaction is calculated to be exothermic (Δ*E* = −32.0 kcal mol^−1^), and the energy of the MECP is +25.2 kcal mol^−1^ with respect to the separate reactants, a much higher value than the experimental activation barrier (Δ*H*^≠^_exp_ 5.0 kcal mol^−1^ (ref. 17)). Geometry optimization starting at the MECP leads to reactants and products respectively on the triplet and singlet surface. The TS state optimized with SOC (red line) lies slightly lower in energy, and it is straightforward to obtain a free energy Δ*G*^≠^ = 31.6 kcal mol^−1^ = Δ*H*^≠^ − T*Δ*S*^≠^ (298.15 K) = 22.5 − (−9.1) kcal mol^−1^. Whereas the MECP approach has been recently applied to the study of O_2_ addition to an iridium complex^28^ and of Pd(0) oxidation through formation of a cyclic peroxo complex,^29^ to the best of our knowledge the computation of such a ‘spin-forbidden’ adiabatic TS with a SOC Hamiltonian has never been done before. The frequency calculation reveals only one imaginary frequency, corresponding to the stretching of the Au–H and O–O bonds. Both the MECP and TS structures show significant O–H bonding, Au–H bond breaking, and O–O bond lengthening. Interestingly, for the Pd analogues, the hydrogen abstraction mechanism occurs in the triplet PES with a pure spin TS.^3^ In our study the energy on the triplet PES goes uniformly uphill on decreasing the O–H distance (see Fig. S1 in the ESI†), not allowing location of a pure triplet spin TS, and thus the computation of the MECP is mandatory in order to find a reactive path. However, since the MECP is not a stationary point, the use of the new methodology consisting of the computation of the TS on an adiabatic SOC PES is crucial both for lowering the barrier and for the Δ*G*^≠^ evaluation.

Analogously, the second considered mechanism (Fig. 1b) has been studied starting with triplet and closed-shell singlet potential energy surface scans (see Fig. S2 in the ESI†). We were unable to find any Au(ii) superoxo adducts in this way, and the scans led to high energy regions. The Au(ii) oxidation state is not typically stable,^30^ consistent with these observations. Accordingly this mechanism has not been considered further.

**Fig. 4:**
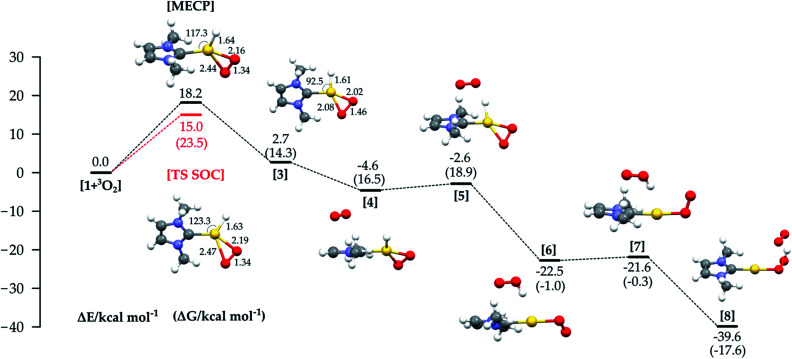
Reaction energy profiles and structures for the oxidative addition/reductive elimination mechanism (the red line shows the energy of the TS optimized with SOC). Δ*G* values are shown in brackets. All energy values (in kcal mol^−1^) refer to the energy of the isolated reactants taken as zero. For structures 4 to 8, the reactants are 1 + two isolated molecules of ^3^O_2_.

In Fig. 4 the energy profile with all the structures involved in the third path (Fig. 1c) is shown. An MECP with significant Au–O bonding to both oxygen atoms is found; this structure also involves significant bending of the initially linear L–Au–H moiety.

The Au–H bond is reactant-like, while the O–O bond is significantly stretched. The MECP lies 18.2 kcal mol^−1^ higher in energy than the separate reactants, lower than the MECP for the hydrogen abstraction mechanism, but still much higher than the experimental Δ*H*^≠^. Optimization from the MECP on the triplet surface leads to reactants, while singlet optimization leads to intermediate 3, lying just above the reactants in energy (but more in free energy).

The MECP for this mechanism was located also using the full experimental system, with the obtained relative energy and structure similar to those obtained with the model (see Fig. S3 in the ESI†). Use of the ZORA SOC method allowed us to locate an adiabatic TS corresponding to the MECP for the model system, with a structure and energy similar to that of the MECP. The calculated imaginary frequency corresponds as expected to the stretching of the two Au–O bonds and the bending of the H–Au–C(carbene) angle.

The calculated activation parameters are Δ*H*^≠^ +14.5 and −T*Δ*S*^≠^ (298.15 K) +9.0 kcal mol^−1^, summing to Δ*G*^≠^ + 23.5 kcal mol^−1^. The Δ*G*^≠^ is in very good agreement with the experimental value (+22.9 kcal mol^−1^), though the enthalpic and entropic contributions agree less well. This may be due to enthalpy–entropy compensation in the solvation free energies: while calculated Δ*G*_sol_ values computed with a continuum model are very small for all species (and are not included by default here, see ESI† for values), the continuum model does not yield separate values for Δ*H*_sol_ and Δ*S*_sol_, so it is *a priori* not possible to estimate the activation entropy and enthalpy in solution when using COSMO. We note that it is known that while entropy and enthalpy of solvation can both make contributions of the same sign to Δ*G*_sol_,^31^ more typical behavior involves compensation, whereby Δ*H*_sol_ and −*T*Δ*S*_sol_ approximately cancel each other out.

We could not find an intramolecular pathway for the recombination of 3 to the product, and instead intermolecular routes, with various partners, were considered. Concerning the Au(i)–H reactivity, solvent, counterion and other additives have been considered.^9,10*b*^ For Pd analogues, the recombination occurs through Pd–O protonolysis carried out by a previously eliminated HX^3^; in our study the best route involved an additional O_2_ molecule (the oxygen is used in large excess experimentally), as shown in Fig. 4, through species 4 to 8. This pathway lies on the triplet PES due to the inclusion of the additional dioxygen molecule. Initial binding of O_2_ to form 4 is slightly uphill in free energy, due to entropic contributions.

From 4, the hydrogen abstraction performed by dioxygen occurs through TS 5 to yield 6, with a calculated activation free energy of only 4.6 kcal mol^−1^ (with respect to 3), and a significant release of free energy. A detailed analysis of the electronic structure for 6 shows one α-spin unpaired electron residing on the unsaturated oxygen of the OOH moiety and the other on the unsaturated oxygen atom of the LAuOO fragment, thus suggesting a diradical intermediate. The final hydrogen transfer is straightforward with a barrier of 0.7 kcal mol^−1^ (TS 7), leading to the thermodynamically very stable product 8 (−17.6 kcal mol^−1^).

This recombination path does not affect the reaction rate, since the bottleneck for the oxidative addition/reductive elimination mechanism is the formation of the MECP in the oxidative addition step.

Very interestingly, our result points out a different gold hydride–O_2_ interaction with respect to the palladium hydride–O_2_ interaction, the latter preferably proceeding through the hydrogen abstraction mechanism.^3^

Mechanism 1c also accounts for the experimental non-reactivity of LAuCH_3_.^17^ Calculations show a similar energy profile to that obtained with LAuH to form a cyclic peroxo species (Δ*E*^≠^ = 19.3 kcal mol^−1^, Δ*E* = 4.9 kcal mol^−1^). However, the CH_3_ abstraction in the following recombination process using O_2_ is unfeasible and the metastable intermediate Au(iii) species will instead revert to reactants (see Fig. S4 in the ESI†). These results not only support the oxidative addition/O_2_ recombination mechanism we found, but also can give the reason why Au(i) complexes (except hydride) are inert toward oxygen as frequently observed in catalytic application.^32^ Very importantly, we find that oxidative addition to Au(i) occurs directly from LAuX (similarly to oxidative addition to L_2_Pd complexes), although it was claimed to be infeasible for gold(i).^16^ For X = alkyl (CH_3_) – and not for X = H – alkyl abstraction in the following recombination process using O_2_ is not feasible, preventing the metastable intermediate Au(iii) species from evolving to products. These findings, contrary to the general belief that LAuX oxidation by O_2_ does not occur, pave the way for a completely new field where X, different from carbon, could be used for Au(i)/Au(iii) oxidation reactions.

**Fig. 5:**
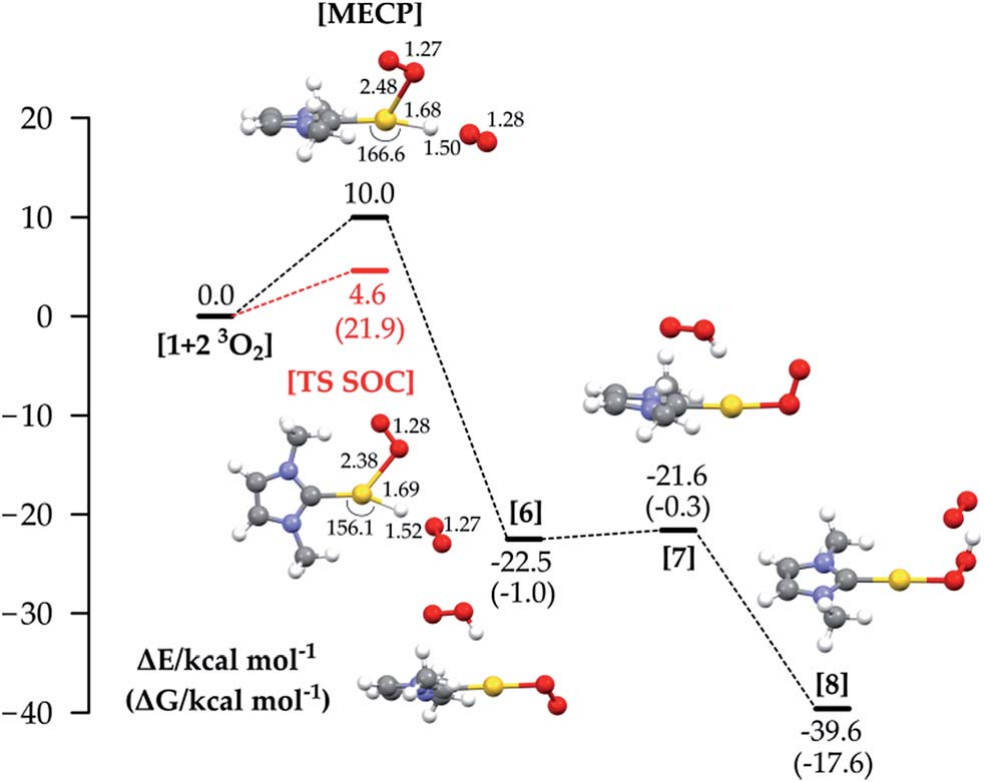
Reaction energy profiles and structures for the mechanism involving two molecules of O_2_ (the red line shows the energy of the TS optimized with SOC). Δ*G* values are shown in brackets. All energy values (in kcal mol^−1^) refer to the energy of the isolated reactants (1 + ^3^O_2_ + ^3^O_2_) taken as zero.

We finally analysed a hypothetical mechanism which includes two O_2_ molecules from the beginning. This allows us also to explore a route that combines the properties of mechanisms 1a and 1b. The energy profile for this path is shown in Fig. 5: the reaction starts with LAuH 1 and two isolated molecules of triplet oxygen. The reaction could in principle involve quintet, triplet or singlet surfaces in the absence of SOC. By comparison with the SOC calculations, we found that the most relevant states were the quintet and triplet, and the reaction passes through a MECP between the quintet and triplet PESs, whose energy is +10 kcal mol^−1^. The reaction then proceeds through the diradical intermediate 6, which with a very low barrier gives the hydroperoxide. We can see that in the MECP the C(carbene)-Au-H angle is bent to 166.6°, the O–H distance is 1.50 Å, and the O–Au distance is 2.48 Å. The adiabatic SOC TS has an imaginary frequency corresponding to simultaneous Au–H, Au–O and H–O bond stretching, and C(carbene)–Au–H angle bending. Very interestingly, for this TS we calculated a Δ*H*^≠^ of 4.3 kcal mol^−1^ (Δ*H*^≠^_exp_ 5.0 kcal mol^−1^) and a −T*Δ*S*^≠^ (298.15 K) of 17.6 kcal mol^−1^ (−T*Δ*S*^≠^_exp_ 17.9 kcal mol^−1^), summing to a Δ*G*^≠^ of 21.9 kcal mol^−1^, which is in very good agreement with Δ*G*^≠^_exp_ = 22.9 kcal mol^−1^. In experiments, a rate law with first order dependence on the concentration of 1 and on the partial pressure of O_2_ was found. This argues against the suggested mechanism with two O_2_ molecules, and in favour of mechanism 1c, though we note that only relatively few O_2_ pressures were used for measurements so partial second-order character cannot be completely ruled out. In favour of the mechanism involving two O_2_ molecules, the calculations yield a Δ*G*^≠^ slightly lower than that for mechanism 1c, and notably a −T*Δ*S*^≠^ which is twice the value for mechanism 1c. This result, together with the unexpectedly high experimental entropic activation parameter,^33^ suggests the possibility that a third species (solvent, counterion, or additive) could be involved in the rate-determining step TS of the reaction.

The energies reported above are all based upon *in vacuo* calculations with the BP86-D3-BJ method, and a DZP basis set (TZP on gold). In the ESI,† detailed tests are reported on the effect of changing the basis set or exchange-correlation functional, and of including a continuum solvent treatment. The basis set and solvation have only modest effects and the energy barriers for the three mechanisms follow the same order irrespective of this aspect. With DFT functionals similar to BP86, we also see only small changes. The B3LYP-D3-BJ functional leads to an increase in the barrier heights for mechanism (c) and especially (c) +O_2_. Without carrying out extensive benchmarking that goes beyond the scope of this work, it is difficult to be certain which functional will yield the best results.^34,35^

## Conclusions

In summary, we demonstrated the feasibility under mild conditions (in terms of Δ*G*^≠^) of Au(i)/Au(iii) oxidation by O_2_, at a variance with the Pd(ii)–hydride O_2_ insertion. Inclusion of SOC effects for the TS search (to the best of our knowledge never applied before) has a sizeable impact on lowering the activation barriers and, when the MECP does not reside on pure spin PESs, it has a crucial influence on the reaction path. We found the oxidative addition/recombination to be the kinetically favoured mechanism for the reaction of a carbene–Au(i)–hydride complex with O_2_. A hypothetical mechanism involving two O_2_ molecules is also feasible, suggesting the possibility that a third species (solvent, counterion, or additive) could be involved in the rate determining step of the reaction. The reason why the typical nucleophilic addition on unsaturated systems catalyzed by homogeneous gold(i) complexes are insensitive to O_2_ has been explained: although the O_2_ oxidative addition to Au–alkyl (CH_3_) occurs, the following recombination process using additional O_2_ does not and the metastable intermediate Au(iii) species will revert to reactants. We believe that this study can pave the way for further theoretical and experimental investigations in the field of Au(i)/Au(iii) oxidation reactions, including ligand, additive and solvent effects, as well as X effects in Au–X bonds (where X is different from carbon). From a theoretical point of view, we have shown that our approach which includes spin–orbit coupling within DFT to investigate the spin-switching process during this reaction is highly promising and useful to distinguish between the reaction mechanisms we considered and to demonstrate the preference for the oxidative addition path.

## Supplementary Material

SC-007-C6SC02161A-s001

SC-007-C6SC02161A-s002
