# Supplementary material for: Dioxygen insertion into the gold(i)–hydride bond: spin orbit coupling effects in the spotlight for oxidative addition
Source: Chem Sci. 2016 Jul 25;7(12):7034–9. doi: 10.1039/c6sc02161a (PMC6413884; doi:10.1039/c6sc02161a)
Supplement: SC-007-C6SC02161A-s001 [file SC-007-C6SC02161A-s001.pdf]

Electronic Supplementary Material (ESI) for Chemical Science.
This journal is © The Royal Society of Chemistry 2016

SUPPORTING INFORMATION – XYZ STRUCTURES

Reactants

```
   17
LAuH scalar
C          4.393262   -1.762505    7.081148
C          4.812023   -1.670532    5.790757
N          3.735999   -1.196472    5.061225
C          2.644910   -0.984888    5.852851
N          3.075167   -1.341597    7.097599
C          3.746961   -0.948236    3.626919
Au         0.773863   -0.285601    5.295354
C          2.235546   -1.279403    8.285101
H          5.768119   -1.898692    5.338340
H          4.913089   -2.086372    7.973184
H          2.132661   -2.280441    8.723286
H          2.672256   -0.587056    9.016273
H          1.253295   -0.911260    7.966665
H          3.957667   -1.880615    3.087521
H          2.750618   -0.580133    3.355691
H          4.501864   -0.189326    3.384392
H         -0.682851    0.258578    4.861478
```

```
  17
LAuH SOC
C       4.389227   -1.762564    7.079979
C       4.808536   -1.669984    5.789660
N       3.732955   -1.195894    5.059719
C       2.641387   -0.985025    5.851187
N       3.070248   -1.344873    7.095744
C       3.744014   -0.945433    3.625805
Au      0.775661   -0.285925    5.296031
C       2.231626   -1.275870    8.283583
H       5.764256   -1.899730    5.337293
H       4.908377   -2.087919    7.971855
H       2.182869   -2.261687    8.763225
H       2.633037   -0.531670    8.983582
H       1.231162   -0.971646    7.954483
H       3.975794   -1.872605    3.086201
H       2.741148   -0.597271    3.352101
H       4.483894   -0.170750    3.386783
H      -0.681376    0.259159    4.863910
```

Interatomic distance O2 scalar= 1.235 Å

Interatomic distance O2 SOC= 1.235 Å

Product

```
   19
LAuOOH scalar
C        -0.477504    0.939769    4.089054
C        -0.760308    0.921288    2.760313
N         0.057707   -0.038448    2.191407
C         0.854641   -0.630132    3.132372
N         0.508505   -0.007963    4.299854
C         0.077434   -0.382263    0.778179
Au        2.196879   -2.049090    2.854432
C         1.108234   -0.311215    5.588958
H        -0.886198    1.538654    4.891997
H        -1.464393    1.500641    2.178042
H         0.336089   -0.653797    6.289498
H         1.839346   -1.111284    5.428215
H         1.616813    0.577333    5.984325
H        -0.909859   -0.748262    0.469024
H         0.363980    0.494766    0.184010
H         0.821321   -1.175485    0.642995
O         3.573227   -3.482801    2.662746
O         3.614890   -3.858434    1.190555
H         4.311476   -4.550779    1.269828
```

Hydrogen abstraction mechanism

```
  19
MECP scalar
      N      0.9928576     -0.4992997      3.1689648
      C      0.9803817      0.1675716      4.3818120
      C     -0.1557383      0.9142829      4.4033807
      N     -0.8055822      0.6818941      3.2040869
      C     -0.1074079     -0.1929789      2.4290671
      C     -2.0691249      1.2921973      2.8103552
      Au    -0.6308949     -0.8629342      0.5628989
      C      2.0410856     -1.4125165      2.7350961
      O     -2.9078689     -1.3162569     -2.1676584
      O     -1.8660614     -1.9849536     -1.6433648
      H      1.7686683      0.0555462      5.1146775
      H     -0.5510617      1.5812837      5.1582191
      H      2.1056177     -2.2590522      3.4300397
      H      1.7692194     -1.7731188      1.7371048
      H      2.9994937     -0.8798426      2.6908793
      H     -2.8446567      1.0432614      3.5451690
      H     -1.9468964      2.3800303      2.7340319
      H     -2.3419959      0.8803152      1.8323865
      H     -1.0718177     -1.2631355     -1.1744368
```

```
   19
TS SOC
  N           0.989451629    -0.513158171     3.192442944
  C           1.025491766     0.217124868     4.368637460
  C          -0.106144000     0.973444047     4.392762368
```

N	-0.803633968	0.681286268	3.232512817
C	-0.138723609	-0.238772254	2.479419063
C	-2.079325882	1.275902324	2.853125039
AU	-0.634322754	-0.899651166	0.603592733
C	2.005227821	-1.469345358	2.771129502
O	-2.810715454	-1.575504877	-2.192774067
O	-1.588714867	-2.004264505	-1.766540307
H	1.840289231	0.137270447	5.077197412
H	-0.468991725	1.682739184	5.126037410
H	2.007501415	-2.336402450	3.444503811
H	1.750450357	-1.792543138	1.754709804
H	2.989815278	-0.984755505	2.768903064
H	-2.837185163	1.047903950	3.613665912
H	-1.964600958	2.361629351	2.740998989
H	-2.374891429	0.836578456	1.892994401
H	-1.051097402	-1.174744610	-1.318628388

Oxidative addition mechanism

19

MECP scalar

C	-0.6439697	0.6740444	4.0637058
C	-0.8451890	0.8530531	2.7312016
N	0.0857795	0.0634892	2.0790228
C	0.8724467	-0.6107496	2.9684884
N	0.4073001	-0.2166686	4.1906823
C	0.2159445	-0.0515508	0.6356450
Au	2.1580988	-2.1566720	2.5812259
O	4.5192289	-1.5538969	2.7204216
O	4.2077255	-2.8264531	2.4176978
C	0.9370264	-0.7051591	5.4533720
H	-1.1471420	1.1011514	4.9209873
H	-1.5559904	1.4706768	2.1984840
H	0.2233518	-1.3957851	5.9211722
H	1.8692032	-1.2377626	5.2393345
H	1.1450955	0.1404736	6.1201784
H	-0.7122797	-0.4493582	0.2058777
H	0.4517164	0.9273153	0.1994700
H	1.0348120	-0.7498235	0.4352231
H	1.4971395	-3.6226518	2.2695255

19

TS SOC

N	0.628222	-0.085118	4.150344
C	-0.425739	0.808901	4.110112
C	-0.891903	0.804881	2.832275
N	-0.116991	-0.099673	2.130093
C	0.834202	-0.659258	2.929823
C	-0.265108	-0.409085	0.716500
AU	2.076540	-2.238598	2.496476
O	4.102840	-2.982520	2.856796
O	4.279562	-1.842500	3.534481
C	1.404038	-0.410732	5.338251
H	-0.750125	1.365099	4.979767

H	-1.694033	1.364484	2.369385
H	0.871515	-1.155397	5.943958
H	2.367356	-0.822526	5.013274
H	1.571344	0.502596	5.921458
H	-1.331665	-0.464522	0.468152
H	0.228519	0.357844	0.105852
H	0.208881	-1.382666	0.542953
H	1.578794	-3.582934	1.710554

19

Struc 3 scalar

C	-0.485712	0.677233	4.138847
C	-0.905920	0.767404	2.849169
N	-0.107951	-0.087509	2.109064
C	0.806278	-0.705478	2.903738
N	0.564356	-0.221953	4.154497
C	-0.198648	-0.256262	0.664967
Au	2.229327	-2.081692	2.479456
O	3.935281	-1.916808	3.663993
O	3.980259	-3.053098	2.742279
C	1.295069	-0.624611	5.353684
H	-0.839821	1.167546	5.035674
H	-1.689334	1.360805	2.397185
H	0.673931	-1.300557	5.955121
H	2.213951	-1.143964	5.046739
H	1.552411	0.270899	5.931580
H	-1.248523	-0.398854	0.382353
H	0.216377	0.625105	0.159222
H	0.382903	-1.144562	0.397469
H	1.310712	-2.789857	1.362895

21

Struc 4 scalar

N	0.060738	-0.995938	3.357750
C	-1.095268	-0.475391	2.805792
C	-0.853645	-0.307104	1.479456
N	0.439161	-0.735764	1.249971
C	1.011099	-1.167075	2.400747
C	1.072953	-0.739273	-0.061731
Au	2.848956	-1.932823	2.748223
O	4.570481	-2.270285	3.778005
O	3.799786	-1.322042	4.522991
C	0.221448	-1.359092	4.761757
H	-1.978889	-0.279178	3.397608
H	-1.486863	0.056292	0.681956
H	-0.233712	-2.340455	4.946115
H	1.292424	-1.399396	4.994693
H	-0.258063	-0.594795	5.383882
H	0.425888	-1.282512	-0.761352
H	1.231899	0.293213	-0.397782
H	2.034560	-1.252042	0.027640
H	2.744516	-2.802828	1.412992
O	-0.801482	-3.421196	0.720442
O	-1.398081	-2.881214	-0.255592

21

Struc 5 scalar

N	0.329431	-0.380408	3.875975
C	-0.809862	0.352087	3.598606
C	-0.953359	0.352633	2.246490
N	0.093814	-0.389115	1.730476
C	0.892338	-0.846661	2.727829
C	0.338846	-0.586330	0.305645
Au	2.532715	-2.011568	2.649250
O	4.380302	-2.742421	3.079187
O	4.259696	-1.561418	3.864645
C	0.840269	-0.658632	5.214048
H	-1.412288	0.800771	4.376939
H	-1.697785	0.814169	1.611796
H	0.298466	-1.505824	5.653318
H	1.906309	-0.904743	5.135432
H	0.716503	0.235489	5.835559
H	-0.609001	-0.824397	-0.189770
H	0.779671	0.323058	-0.122688
H	1.024008	-1.429607	0.181442
H	1.758642	-2.975111	1.603004
O	0.833690	-4.082030	0.526346
O	0.758151	-3.638522	-0.667691

21

Struc 6 scalar

N	-0.062524	-0.867962	3.541942
C	-1.218499	-0.268989	3.080796
C	-1.007826	0.029568	1.771241
N	0.271079	-0.389982	1.467190
C	0.860440	-0.960415	2.548955
C	0.854971	-0.356461	0.133270
Au	2.646829	-1.848772	2.641996
O	4.480116	-2.762647	2.794584
O	4.929287	-2.520328	4.038719
C	0.113482	-1.403761	4.884818
H	-2.077966	-0.104903	3.716686
H	-1.651416	0.496965	1.038350
H	-0.507036	-2.300697	5.006730
H	1.168213	-1.669915	5.008314
H	-0.162427	-0.641507	5.623313
H	0.687033	0.631179	-0.312558
H	1.929466	-0.541724	0.229312
H	0.400761	-1.144731	-0.480851
H	1.379279	-3.280324	1.478714
O	0.733037	-3.700120	0.808702
O	-0.497740	-3.243509	1.203276

21

Struc 7 scalar

N	-0.106893	-0.855764	3.489343
C	-1.284945	-0.344649	2.980890
C	-1.062402	-0.086026	1.664898
N	0.245571	-0.441382	1.404837
C	0.840104	-0.937343	2.519455
C	0.847177	-0.464020	0.078303
Au	2.675210	-1.707682	2.677170
O	4.539255	-2.552840	2.846758

O	4.407071	-3.806680	3.314136
C	0.063483	-1.345101	4.850315
H	-2.165883	-0.204432	3.592625
H	-1.714366	0.316478	0.901526
H	-0.490766	-2.284289	4.973830
H	1.130572	-1.528227	5.012595
H	-0.294284	-0.589654	5.560015
H	0.566124	0.446821	-0.462937
H	1.934640	-0.503271	0.197440
H	0.503387	-1.358047	-0.458419
H	1.529692	-3.382074	1.755164
O	0.906197	-3.952637	1.187969
O	-0.336216	-3.421236	1.422785

21

Struc 8 scalar

N	0.319297	0.251358	3.554064
C	-0.892266	0.771760	3.137000
C	-1.240360	0.095059	2.010282
N	-0.229953	-0.815005	1.762101
C	0.743754	-0.735370	2.713183
C	-0.220534	-1.771509	0.661066
Au	2.382864	-1.842164	2.865343
O	4.078568	-2.926424	3.085172
O	4.046633	-4.140835	2.277980
C	1.056500	0.702452	4.725955
H	-1.391873	1.571577	3.666934
H	-2.107147	0.184477	1.369292
H	0.433196	0.589258	5.621773
H	1.948639	0.073773	4.816595
H	1.355384	1.750586	4.597922
H	-0.660457	-1.300515	-0.225445
H	0.816391	-2.049237	0.452724
H	-0.789061	-2.668946	0.936458
H	3.637215	-3.833262	1.416614
O	2.873776	-3.318755	-0.074076
O	1.986438	-4.228171	-0.169197

2 O_2 molecules mechanism

21

MECP scalar

N	1.7332698	0.9943314	0.1934017
C	3.0823735	0.6967634	0.2591880
C	3.2048092	-0.6216835	-0.0537807
N	1.9259642	-1.0887661	-0.3006022
C	1.0021663	-0.1023810	-0.1441422
C	1.5894769	-2.4618378	-0.6502880
Au	-1.0629715	-0.2361697	-0.3060951
C	1.1475841	2.2926066	0.4997473
H	3.8285223	1.4371116	0.5171793
H	4.0782639	-1.2564387	-0.1229578
H	1.6793824	3.0749939	-0.0552839
H	0.0989422	2.2624975	0.1864998
H	1.1959260	2.4785106	1.5799326

H	2.1863822	-2.7823345	-1.5128997
H	1.7721395	-3.1220812	0.2071761
H	0.5249325	-2.4803657	-0.9095219
H	-2.6608605	-0.4013732	-0.8170737
O	-4.0534742	-0.6618368	-1.3113637
O	-4.7842101	0.3833864	-1.2327628
O	-2.4020661	0.2957561	1.7141378
O	-1.5934838	0.8828995	2.5052394

21

TS SOC

C	0.963509967	0.051503984	-0.048503853
N	1.851566352	0.935505710	0.484428089
C	3.153958562	0.526322889	0.264563093
C	3.085244347	-0.643019520	-0.425639270
N	1.742312306	-0.915299196	-0.606479195
C	1.484752016	2.159937255	1.183247042
C	1.218948298	-2.085830689	-1.297056543
AU	-1.104221547	0.106160741	-0.053414954
O	-2.617828568	1.643495478	0.959228473
O	-1.870849356	2.504912050	1.549490086
O	-4.014091808	-1.063007446	-0.706071870
O	-4.331283552	-1.056128909	-1.940693648
H	4.006787727	1.094454975	0.612565199
H	3.865411778	-1.294886374	-0.796544937
H	1.839049036	3.028985590	0.614632329
H	0.391994322	2.195773324	1.265250624
H	1.927897226	2.154461897	2.186768056
H	1.601481169	-2.110777672	-2.324878086
H	1.507700636	-2.996478426	-0.757532630
H	0.126507169	-1.994159385	-1.310152678
H	-2.663093059	-0.416069769	-0.445826770

XYZ OF STRUCTURES IN THE S.I.

19

Fig. S1 left- 1.35 singlet

N	1.089012	-0.612094	3.017696
C	1.099998	0.110730	4.196733
C	-0.060385	0.819411	4.233124
N	-0.750142	0.515820	3.074473
C	-0.050874	-0.372979	2.309673
C	-2.053569	1.060166	2.708957
Au	-0.629400	-1.104883	0.582947
C	2.147582	-1.508311	2.574455
O	-2.428554	-0.682935	-1.225227
O	-1.405849	-1.698876	-1.263153
H	1.922142	0.061188	4.897952
H	-0.448110	1.505036	4.974672
H	2.322323	-2.278949	3.335804
H	1.799910	-1.972121	1.642906
H	3.066392	-0.937963	2.388158

```
H        -2.280202    0.730815    1.686608
H        -2.820076    0.680950    3.396523
H        -2.014626    2.155687    2.745474
H        -1.883017   -2.905702   -0.891154
```

19

Fig. S1 right – 1.35 triplet

```
N         1.884836    0.205631    2.565903
C         2.061339    0.645426    3.866375
C         0.817449    0.810993    4.390733
N        -0.079117    0.466796    3.393893
C         0.563868    0.088664    2.254055
C        -1.528675    0.498303    3.529783
Au       -0.277438   -0.528672    0.448950
C         2.962427   -0.099464    1.635293
O        -2.675291   -1.590354   -2.294476
O        -1.400862   -1.440802   -2.252275
H         3.040055    0.800881    4.301178
H         0.500104    1.138979    5.371973
H         3.580845   -0.913578    2.034094
H         2.497992   -0.414633    0.693893
H         3.573689    0.795958    1.465652
H        -1.844078   -0.190874    4.323379
H        -1.859727    1.519401    3.757697
H        -1.952094    0.177183    2.571254
H        -0.973047   -1.038235   -1.036786
```

19

Fig. S2 – singlet 1.8

```
C         2.036103   -1.071919   -0.455808
N         1.325256    0.079590   -0.738135
C         1.570588    1.048773    0.191816
N         2.443833    0.469045    1.062209
C         2.738228   -0.828809    0.681978
C         0.466194    0.256746   -1.904649
Au        0.742346    2.910511    0.132153
C         2.958954    1.104312    2.266829
H         3.408362   -1.459165    1.250936
H         1.982433   -1.953135   -1.080984
H         4.053578    1.032124    2.279981
H         2.658308    2.156195    2.243167
H         2.534949    0.617906    3.154980
H        -0.161738   -0.633903   -2.025564
H        -0.170157    1.136623   -1.741937
H         1.081251    0.412304   -2.800648
H         2.060248    3.553741    0.787415
O        -0.378621    4.279288   -0.199358
O        -1.153537    3.127947   -0.711861
```

69

MECP scalar oxidative addition_ experimental carbene

```
    C    -3.0082233      4.6314585     -0.9322436
    C    -3.0454607      4.6020373      0.4669129
    C    -3.1805610      5.7485885      1.2590885
    C    -3.2910698      6.9723267      0.5966275
```

C	-3.2636677	7.0361083	-0.7926438
C	-3.1259990	5.8779669	-1.5499707
N	-2.8729989	3.3391161	1.1207660
C	-3.8559276	2.3825245	1.3311871
C	-3.2493004	1.3332742	1.9480648
N	-1.9155552	1.6831802	2.1003092
C	-1.6629362	2.9275473	1.6007810
C	-0.8979116	0.8796374	2.7095328
C	-0.6816353	1.0142345	4.0852063
C	0.3248107	0.2312243	4.6542631
C	1.0819844	-0.6332186	3.8712127
C	0.8578023	-0.7241475	2.5013111
C	-0.1360308	0.0400802	1.8877904
C	-1.4758214	1.9831853	4.9316005
C	-2.2731074	1.2428885	6.0111877
C	-0.3672279	-0.0437061	0.3956431
C	0.9249251	0.2144869	-0.3836412
Au	0.1386915	3.8369758	1.4258190
C	-3.2548475	5.6725162	2.7676177
C	-2.3242045	6.6788698	3.4453783
C	-2.7941646	3.3793630	-1.7542685
C	-3.8757114	3.2180947	-2.8270085
C	-4.7087378	5.8606956	3.2223016
C	-1.3887848	3.3805707	-2.3674681
C	-0.9844962	-1.3969961	0.0227706
C	-0.5584588	3.0468933	5.5435157
H	-4.8812330	2.5413046	1.0222842
H	-3.6332533	0.3838678	2.2989365
H	1.0992798	3.5803244	0.1216267
H	-3.3925119	7.8894091	1.1760692
H	-3.3437399	8.0019118	-1.2909900
H	-3.0943073	5.9455376	-2.6374794
H	-3.7332664	2.2724548	-3.3710739
H	-4.8825914	3.2193963	-2.3831277
H	-3.8292531	4.0352358	-3.5626839
H	-2.9294123	4.6676252	3.0722054
H	-1.2208013	2.4517641	-2.9339368
H	-1.2700066	4.2308090	-3.0580900
H	-0.6204171	3.4636437	-1.5848597
H	1.4741600	-1.3901097	1.8973740
H	1.8660012	-1.2336677	4.3319260
H	0.5267608	0.3090259	5.7225026
H	-1.0800406	0.7464558	0.1189023
H	0.7182775	0.2187609	-1.4642854
H	1.3546152	1.1877182	-0.1067939
H	1.6708003	-0.5712925	-0.1874109
H	-1.1857094	-1.4423576	-1.0583081
H	-0.2958526	-2.2181951	0.2769494
H	-1.9279144	-1.5697057	0.5623111
H	-2.1931089	2.4979138	4.2757235
H	-2.8818403	1.9537741	6.5899013
H	-2.9401957	0.4871109	5.5699412
H	-1.5968206	0.7293774	6.7119935
H	-1.1528621	3.7680750	6.1247758
H	0.1724594	2.5844723	6.2254544
H	-0.0061618	3.5970885	4.7676271
H	-4.7838942	5.7631562	4.3159560

H	-5.0713352	6.8620788	2.9404683
H	-5.3744146	5.1178877	2.7571317
H	-2.3804719	6.5591684	4.5377021
H	-1.2796901	6.5241731	3.1383127
H	-2.6167813	7.7137284	3.2104758
H	-2.8609212	2.5124671	-1.0806506
O	1.5984715	5.1616807	2.2923372
O	0.7480775	5.3209232	3.3171180

22

Fig. S4 left- MECP oxidative addition – LAuCH3

N	0.0749909	0.0752321	2.0846916
C	0.8715044	-0.5866198	2.9770907
N	0.3967339	-0.1970617	4.1990634
C	-0.6646869	0.6810903	4.0675732
C	-0.8658093	0.8552934	2.7348598
Au	2.1963086	-2.0735881	2.5934301
O	4.6510160	-1.6396559	2.7095530
O	4.1670452	-2.8601564	2.4022839
C	0.9291080	-0.6768292	5.4644662
C	0.2091435	-0.0357934	0.6410770
C	1.3556045	-4.0166698	2.1737319
H	-1.1735468	1.1075037	4.9218420
H	-1.5809898	1.4661742	2.2005239
H	0.2219483	-1.3746760	5.9319149
H	1.8697618	-1.1970292	5.2549988
H	1.1210705	0.1731524	6.1311532
H	-0.7234719	-0.4198425	0.2083650
H	0.4542949	0.9430288	0.2091602
H	1.0224741	-0.7406332	0.4387672
H	2.1805781	-4.7160423	1.9898500
H	0.7684000	-4.3204625	3.0505315
H	0.7065464	-3.9311016	1.2926069

22

Fig. S4 middle – Product oxidative addition – LAuCH3

N	-0.103858	-0.148299	2.110016
C	0.832609	-0.699508	2.927639
N	0.576959	-0.170027	4.155514
C	-0.506681	0.688349	4.107075
C	-0.933162	0.707158	2.816235
Au	2.268256	-2.071785	2.565268
O	4.154009	-1.638316	3.359566
O	4.089130	-2.938667	2.694609
C	1.340783	-0.497926	5.355339
C	-0.181055	-0.372825	0.672830
C	0.964537	-3.421804	1.587678
H	-0.872742	1.208294	4.982131
H	-1.735677	1.255939	2.341671
H	0.838903	-1.300187	5.911204
H	2.340638	-0.834001	5.048211
H	1.425911	0.399481	5.978685
H	-1.155184	-0.806637	0.415660
H	-0.036291	0.578293	0.144300
H	0.615410	-1.070053	0.400043

```
H         1.176511   -4.419602    1.995793
H        -0.092916   -3.174186    1.751177
H         1.190980   -3.423260    0.511189
```

24

Fig. S4 right – Max. Rearrang. – LAuCH3

```
C        -1.073508    0.389665    3.047430
N        -0.173188   -0.343417    2.293058
C         0.814732   -0.844860    3.078286
N         0.527740   -0.405931    4.329691
C        -0.633467    0.342828    4.332924
C        -0.253456   -0.517272    0.849622
Au        2.332365   -2.086448    2.592617
C         0.947595   -3.603067    1.943615
C         1.289255   -0.781135    5.514522
O         4.351308   -1.500651    2.808751
O         4.216298   -2.847870    2.346456
H        -1.041566    0.771861    5.238012
H        -1.929883    0.881816    2.606521
H         0.895951   -1.724038    5.915032
H         2.338058   -0.911176    5.225681
H         1.208782    0.020548    6.257068
H        -1.204860   -0.995788    0.586720
H        -0.167530    0.458281    0.353564
H         0.576711   -1.157984    0.539989
H         1.253495   -4.645816    2.077067
H        -0.132379   -3.431336    1.972622
H         1.211664   -3.411865    0.860873
O         0.627176   -3.886537    3.794824
O        -0.358128   -3.289176    4.353940
```

17

Fig. S5 – LAuH SOC TZP

```
C         4.396210   -1.768036    7.083234
C         4.816660   -1.673693    5.790582
N         3.738515   -1.196567    5.057957
C         2.647184   -0.985904    5.853339
N         3.074349   -1.344839    7.101022
C         3.747996   -0.944302    3.621073
Au        0.773230   -0.285327    5.295202
C         2.232819   -1.275557    8.290739
H         5.774576   -1.904231    5.341281
H         4.918828   -2.095330    7.973363
H         2.164224   -2.263138    8.763273
H         2.642782   -0.550293    9.004809
H         1.238362   -0.949873    7.966376
H         3.973905   -1.868895    3.075500
H         2.748529   -0.588621    3.347591
H         4.492933   -0.177175    3.375359
H        -0.684550    0.257968    4.860887
```

19

Fig. S5- TS SOC TZP Hydrogen Abstraction

```
N         0.995008074    -0.502706378     3.201111790
C         1.034253256     0.238114134     4.374383535
```

C	-0.105048335	0.985617475	4.402812754
N	-0.810708284	0.680555955	3.247114329
C	-0.142291372	-0.240417240	2.496171747
C	-2.096361563	1.262979011	2.871063572
AU	-0.667508551	-0.949004867	0.636351960
C	2.021186816	-1.447907323	2.770578873
O	-2.791014797	-1.288145788	-2.327654722
O	-1.747410490	-1.971231197	-1.784246029
H	1.855024359	0.172281739	5.078075672
H	-0.466602352	1.695849243	5.136436816
H	2.073061236	-2.294098420	3.466727713
H	1.739359883	-1.807539488	1.774753730
H	2.994328155	-0.945015723	2.720714031
H	-2.850913889	1.031080497	3.632436544
H	-1.998143677	2.349630900	2.760848507
H	-2.390803323	0.821850261	1.912547817
H	-1.069325303	-1.280309392	-1.325832179

19

Fig. S5- TS SOC TZP Oxidative Addition

N	0.638686	-0.079798	4.155711
C	-0.407396	0.829427	4.118435
C	-0.888943	0.820095	2.843659
N	-0.129997	-0.101017	2.138107
C	0.822634	-0.665029	2.935197
C	-0.294605	-0.418360	0.724489
AU	2.078335	-2.243641	2.484981
O	4.108728	-2.958886	2.896523
O	4.247897	-1.830857	3.598017
C	1.425772	-0.404171	5.341153
H	-0.716670	1.399002	4.985765
H	-1.691256	1.385788	2.387079
H	0.876272	-1.103253	5.983785
H	2.361957	-0.870365	5.012737
H	1.645422	0.515375	5.894973
H	-1.362031	-0.488847	0.487480
H	0.175556	0.352042	0.100875
H	0.193060	-1.382726	0.544815
H	1.638391	-3.579032	1.651245

21

Fig. S5- TS SOC TZP – 2 O_2 molecules

C	0.968018	0.044317	-0.047334
N	1.855618	0.937484	0.475445
C	3.161568	0.531836	0.244094
C	3.092274	-0.645487	-0.437784
N	1.745424	-0.925187	-0.608583
C	1.484436	2.163579	1.176826
C	1.217577	-2.103401	-1.289068
AU	-1.105644	0.107952	-0.049274
O	-2.611300	1.663487	0.966393
O	-1.873184	2.512708	1.587280
O	-4.026085	-1.044516	-0.736474
O	-4.325797	-1.101899	-1.971857
H	4.016640	1.105014	0.580097
H	3.874522	-1.295762	-0.809003
H	1.831193	3.036397	0.610621

H	0.392492	2.196787	1.266708
H	1.933767	2.167200	2.176861
H	1.582176	-2.135094	-2.322498
H	1.519636	-3.013450	-0.757146
H	0.125037	-2.020644	-1.285741
H	-2.664433	-0.412212	-0.451566
