# Supplementary material for: Dioxygen insertion into the gold(i)–hydride bond: spin orbit coupling effects in the spotlight for oxidative addition
Source: Chem Sci. 2016 Jul 25;7(12):7034–9. doi: 10.1039/c6sc02161a (PMC6413884; doi:10.1039/c6sc02161a)
Supplement: SC-007-C6SC02161A-s002 [file SC-007-C6SC02161A-s002.pdf]

Electronic Supplementary Material (ESI) for Chemical Science.
This journal is © The Royal Society of Chemistry 2016

Electronic Supplementary Information

Dioxygen insertion into the gold(I)-hydride bond: spin orbit coupling effects in the spotlight for oxidative addition

Carlo Alberto Gaggioli[a], Leonardo Belpassi[b], Francesco Tarantelli[ab], Daniele Zuccaccia[bc], Jeremy N. Harvey*[d] and Paola Belanzoni*[ab]

[a.] *Department of Chemistry, Biology and Biotechnology, University of Perugia, via Elce di Sotto, 8 – 06123 Perugia (Italy) E-mail: paola.belanzoni@unipg.it*
[b.] *Institute of Molecular Science and Technologies (ISTM) – CNR via Elce di Sotto, 8 – 06123 Perugia (Italy)*
[c.] *Department of Food, Environmental and Animal Sciences, Section of Chemistry, University of Udine, via Cotonificio, 108 – 33100 Udine (Italy)*
[d.] *Department of Chemistry, KU Leuven, Celestijnenlaan 200F, B-3001 Heverlee (Belgium) E-mail: jeremy.harvey@chem.kuleuven.be*

Contents

1. Hydrogen abstraction mechanism……………………………………………….2

2. O_2 metal coordination mechanism……………………………………….4

3. Oxidative addition mechanism…………………………………………….5

4. Reliability of the theoretical method

Basis set effects………………………………………………………………….6

D3-BJ dispersion effects…………………………………………………….8

XC commonly used functional (PBE, BLYP, TPSS, B3LYP, M06)……….9

Solvation (benzene) effects………………………………………………….10

1.Hydrogen abstraction mechanism.

For investigating the hydrogen abstraction mechanism, we performed two potential energy surface scans, one for the triplet and one for the closed shell singlet spin state, using the oxygen-hydrogen (hydride) distance as the reaction coordinate, and optimizing all the other degrees of freedom.

The energy profiles is shown in Fig. S1 (top): the separated reactants, corresponding to a very large O-H distance, interact forming a weakly bound van der Waals complex with an O-H distance of 2.80 Å. Upon closer approach of oxygen to the metal hydride, on the triplet PES, the energy increases constantly, reaching a value of about 23 kcal mol^{-1} for an O-H distance of 1.00 Å. At this point the H is almost abstracted from gold, since the Au-H distance is 2.20 Å compared to 1.62 Å in LAuH. The energy of the isolated LAu and OOH radicals is about 16 kcal mol^{-1} higher, suggesting that at this point on the reaction path an interaction between the LAu and OOH fragments is still present. The singlet profile starts at an energy about 20 kcal mol^{-1} higher than that of the isolated reactants (the splitting between the $^3\Sigma_g$ and $^1\Delta$ states of O_2 is +27.6 kcal mol^{-1} at this level of theory, suggesting that at 2.8 Å an interaction between O_2 and LAuH occurs), and, as oxygen approaches hydrogen, the energy slightly increases until an O-H distance of about 1.50 Å. The energy then slightly decreases till 1.35 Å; from this point a sharp lowering of the energy takes place, leading directly to the product, after significant rearrangement of the relative position of the O_2 fragment and the gold complex. The one-dimensional relaxed scans displayed in Fig. S1 (top) show that at an O-H distance of roughly 1.35 Å the triplet and singlet PES have the same energy, but this is only a lower bound to the energy of the MECP, because the structure of the singlet and triplet complexes, except for the identical O-H distance, are significantly different (Fig. S1 bottom). In Fig. S1 (top) we put also the MECP and TS SOC for comparison (as green and red point respectively).

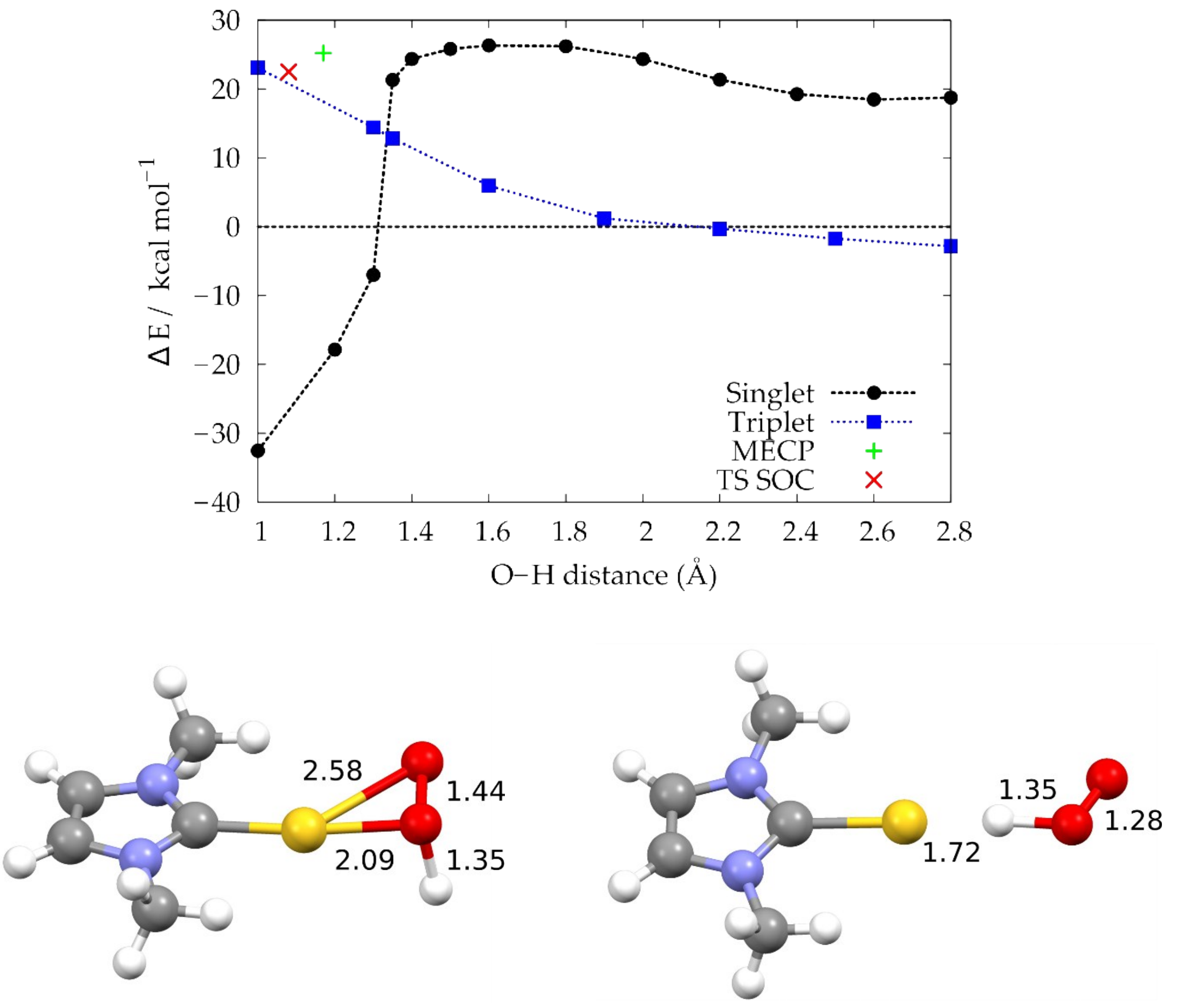

Fig. S1: top) PESs scan (triplet and closed shell singlet spin) using the O-H distance (in Å) as reaction coordinate; MECP and TS SOC are also shown as a green and red point respectively; bottom) geometry of the complex at the O-H distance of 1.35 Å for the singlet (left) and triplet (right) PESs.

2. O_2 metal coordination mechanism.

In Fig. S2 the plot of the PESs scan (triplet and closed shell singlet) using the Au-O distance (in Å) as reaction coordinate for the O_2 metal coordination mechanism is shown. In the Figure the structure of the complex at Au-O = 1.8 Å within the singlet PES is reported. This geometry closely resembles the product of an oxidative addition, and therefore it does not represent the intermediate for the O_2 metal coordination pathway.

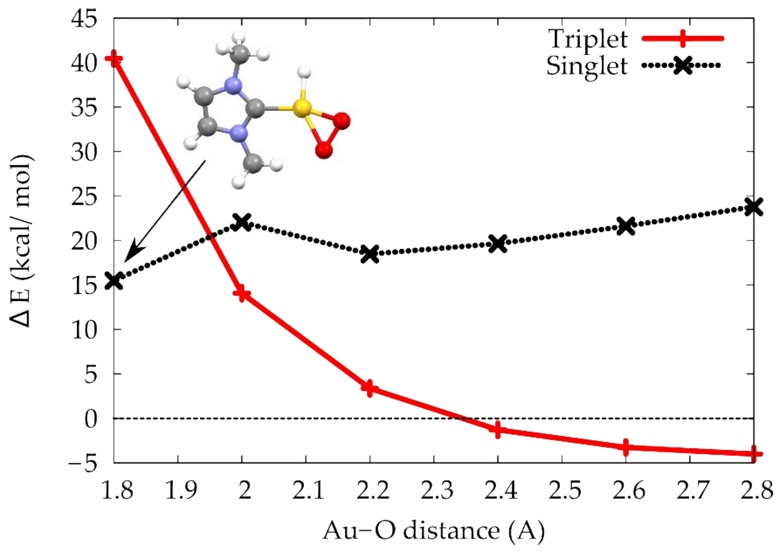

Fig. S2: plot of the PESs scan (triplet and closed shell singlet) using the Au-O distance (in Å) as reaction coordinate. All energy values (in kcal mol^{-1}) refer to the energy of the isolated reactants taken as zero.

3.Oxidative addition mechanism.

In Fig. S3 the MECP for the oxidative addition using the experimental carbene is shown. The energy of this MECP is +17.9 kcal mol^{-1} with respect to the isolated reactants, very close to the energy of the MECP using the computational model (+18.2 kcal mol^{-1}). Also the geometries are very similar.

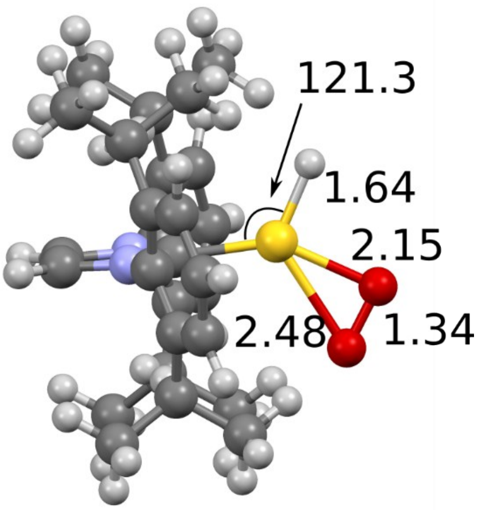

Fig. S3: Geometry of the MECP for the oxidative addition mechanism using the experimental carbene.

In Fig. S4 the MECP for the oxidative addition with $LAuCH_3$ and O_2 as reactants, the product of the oxidative addition (singlet) and the structure with maximum energy in the triplet PES scan for the rearrangement process with O_2 are shown. The oxidative addition is feasible, with a $\Delta E^{\neq}$ of +19.3 kcal mol^{-1} (18.2 kcal mol^{-1} for the MECP with LAuH), and is endothermic with a ΔE of +4.9 kcal mol^{-1} (2.7 kcal mol^{-1} for the MECP with LAuH), while the calculation of the maximum energy point of the rearrangement process gives a ΔE of +34.6 kcal mol^{-1}.

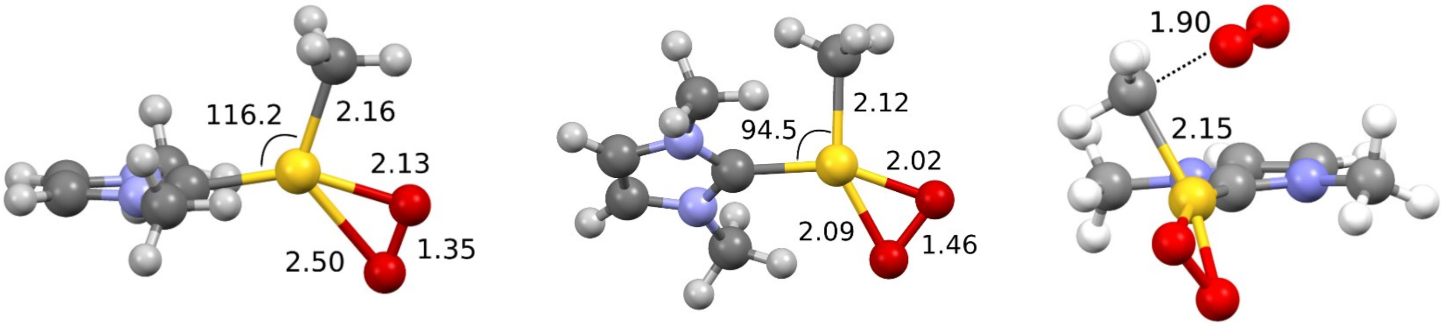

Fig. S4: left) MECP structure for the oxidative addition with $LAuCH_3$, middle) product of the oxidative addition and right) structure corresponding to the maximum energy of the PES scan for the rearrangement path with O_2.

4.Reliability of the theoretical method.

In order to assess the reliability of the level of theory used in this work, we performed additional SOC calculations, aimed at first testing the basis set effects on the geometries and the energies (absolute values of LAuH, O_2, transition states and activation barriers) of the three analyzed mechanisms. We then evaluated the D3-BJ dispersion effects on the energies. We further explored the effects of a few most popular XC functionals on the energies, by comparing 3 GGA (BP86, PBE and BLYP), one meta-GGA (TPSS), one hybrid (B3LYP), and one meta-hybrid (M06) functionals for the three mechanisms studied. Finally, we estimated the solvent effects (benzene) by performing implicit solvation calculations using COSMO model. The results of this study, while assessing the possible error bars one would get by changing any of basis set, exchange-correlation functional, D3-BJ dispersion correction and solvation (benzene) items, both substantiate the reliability of our DFT SOC (BP86-D3-BJ/DZP) level of theory to describe the system for the purpose of this work and confirm the chemical insight we obtain, namely that the oxidative addition is preferred over the H-abstraction mechanism.

Basis set effects.

We performed geometry optimizations using the same computational details as specified in the text (SOC BP86-D3-BJ), but increasing the basis set from DZP to TZP for all atoms. The geometries of the reactants and the transition states (TS SOC) for the three mechanisms, optimized using a DZP and a TZP basis set, are compared in Fig. S5.

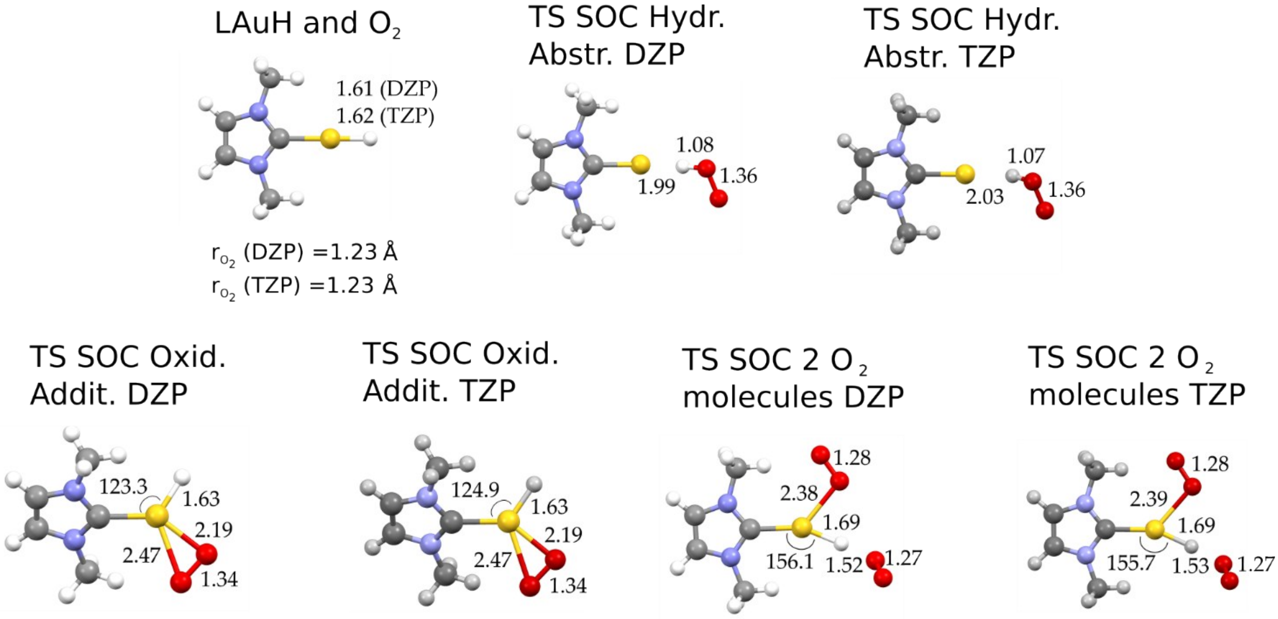

Fig. S5 : Reactants (LAuH and O_2) and transition states (TS SOC) geometries optimized with a DZP and a TZP basis set for the three mechanisms (Hydr. Abstr. = hydrogen abstraction, mechanism a); Oxid. Addit. = oxidative addition, mechanism c); 2 O_2 molecules = mechanism c) with the additional O_2 molecule) .

Figure S5 clearly demonstrates that the basis set quality does not affect the geometries of the species. For the purposes of our paper, the DZP basis set for all atoms (TZP for gold) guarantees a high degree of reliability for geometry optimization calculations.

The energies of all the species optimized with TZP and the single point energies using a TZP and a TZ2P basis set on the DZP optimized structures are compared with the corresponding energies of the species optimized with DZP in Table S1, where activation (electronic) energy barriers are also reported for the 3 considered mechanisms.

Table S1: Energies of optimized DZP and TZP structures (reactants and TS SOC), TZP and TZ2P single point energy calculations on optimized corresponding DZP geometries, and activation (electronic) energy barriers for the three mechanisms a), c) and c) + O_2.

Level of theory	Mechanism	Energy LAuH (Hartree)	Energy O_2 (Hartree)	Energy TS (Hartree)	$\Delta E^{\neq}$ (kcal/mol)
	Hydrog. Abstr.			-3,91872344	22,9
Opt DZP	Oxid. Addit.	-3,60312516	-0,35216473	-3.93139859	15,0
	2 O_2 molec.			-4.29992097	4,7
	Hydrog. Abstr.			-3,93115810	24,3
Opt TZP	Oxid. Addit.	-3,61404599	-0,35576840	-3,94231378	17,3
	2 O_2 molec.			-4,31338785	7,7
	Hydrog. Abstr.			-3,93099011	24,3
Single point TZP	Oxid. Addit.	-3,61394761	-0,35576772	-3,94229561	17,2
	2 O_2 molec.			-4,31276989	8,0
	Hydrog. Abstr.			-3,95201822	25,7
Single point TZ2P	Oxid. Addit.	-3,63057525	-0,36245294	-3,96424689	18,1
	2 O_2 molec.			-4,34023337	9,6

From Table S1 we observe that basis set quality does have an effect on the activation energy barriers. Comparison of the third and fourth sets of data, reporting results of single point energy calculations performed at TZP and at T2ZP level of theory on the geometry optimized at DZP, respectively, with the first set of data shows that the activation energy barriers for the 3 mechanisms increase from DZP to TZP (from +1.4 to +3.3 kcal/mol) but are almost converged from TZP to TZ2P (from +1.4 to +1.6 kcal/mol), with the activation energy barrier for mechanism c) + O_2 being the most sensitive to basis set quality. Interestingly, single point energy calculations performed with TZP basis set on DZP optimized geometries gives activation energy barrier values almost identical to those obtained from TZP optimized geometries.

Importantly, the same activation energy barrier trend, namely $\Delta E^{\neq}$a) > $\Delta E^{\neq}$c) > $\Delta E^{\neq}$c) + O_2 is calculated with all the considered basis sets.

D3-BJ dispersion effects.

The D3-BJ dispersion effects are evaluated by performing single point energy calculations on reactants and TS SOC DZP-optimized structures, using the TZ2P basis set and the BP86 functional with (BP86-D3-BJ) and without dispersion corrections (BP86). Results are compared in Table S2 where activation (electronic) energy barriers for the 3 mechanisms are also shown.

Table S2: D3-BJ dispersion effects on the energies of the reactants and TS SOC (using a TZ2P basis set on the optimized DZP geometries) and activation energy barriers for the three studied mechanisms.

Level of theory	Mechanism	Energy LAuH (Hartree)	Energy O_2 (Hartree)	Energy TS (Hartree)	$\Delta E^{\neq}$ (kcal/mol)
Single point TZ2P with dispersion	Hydrog. Abstr.<br>Oxid. Addit.<br>2 O_2 molec.	-3,63057525	-0,36245294	-3,95201822<br>-3,96424689<br>-4,34023337	25,7<br>18,1<br>9,6
Single point TZ2P without dispersion	Hydrog. Abstr.<br>Oxid. Addit.<br>2 O_2 molec.	-3,60103601	-0,36207276	-3,919726667<br>-3,930258700<br>-4,303440488	27,2<br>20,6<br>13,6

We see that Inclusion of D3-BJ dispersion effects accounts for a non negligible lowering of the activation energy barriers which decrease by 1.5 kcal/mol for the hydrogen abstraction (mechanism a)), by 2.5 kcal/mol for the oxidative addition (mechanism c)) and by 4.0 kcal/mol for the oxidative addition involving two O_2 molecules. Importantly, the same activation energy barrier trend, namely $\Delta E^{\neq}$a) > $\Delta E^{\neq}$c) > $\Delta E^{\neq}$c) + O_2 is calculated also <u>without</u> inclusion of D3-BJ dispersion effects.

XC commonly used functionals (PBE, BLYP, TPSS, B3LYP, M06) effects.

The XC functional effects have been investigated by performing single point energy calculations with a TZ2P basis set and SOC, without inclusion of D3-BJ dispersion effects, on the BP86-D3-BJ DZP optimized reactants and transition state structures for the 3 mechanisms. We selected 5 different popular XC as representative of 2 GGA (PBE and BLYP), 1 meta-GGA (TPSS), 1 hybrid (B3LYP) and 1 meta-hybrid (M06) functional. The results are shown in Table S3, including activation (electronic) energy barriers, and compared with the BP86 outcomes.

Table S3: XC functional effect on the energies of reactants and TS SOC and on the activation energy barriers for the three studied mechanisms. All the calculations have been done without inclusion of D3-BJ dispersion, except for the last row.

XC functional	Mechanism	Energy LAuH (Hartree)	Energy O_2 (Hartree)	Energy TS (Hartree)	$\Delta E^{\neq}$ (kcal/mol)
GGA BP86	Hydrog. Abstr.<br>Oxid. Addit.<br>2 O_2 molec.	-3,60103601	-0,36207276	-3,919726667<br>-3,930258700<br>-4,303440488	27,2<br>20,6<br>13,6
GGA PBE	Hydrog. Abstr.<br>Oxid. Addit.<br>2 O_2 molec.	-3,64301231	-0,36353696	-3,96504543<br>-3,97664702<br>-4,35316754	26,0<br>18,8<br>10,6
GGA BLYP	Hydrog. Abstr.<br>Oxid. Addit.<br>2 O_2 molec.	-3,46308949	-0,34490804	-3,76592333<br>-3,77345381<br>-4,13062832	26,4<br>21,7<br>14,0
MetaGGA TPSS	Hydrog. Abstr.<br>Oxid. Addit.<br>2 O_2 molec.	-35,75621845	-0,3742955	-36,08150759<br>-36,09822390<br>-36,47979971	30,8<br>20,3<br>15,7
MetaHybrid M06	Hydrog. Abstr.<br>Oxid. Addit.<br>2 O_2 molec.	-36,31927638	-0,5400000	-36,80348669<br>-36,81089744<br>-37,36170423	35,0<br>30,4<br>23,6
Hybrid B3LYP	Hydrog. Abstr.<br>Oxid. Addit.<br>2 O_2 molec.	-36,19678471	-0,498715719	-36,63725825<br>-36,64878790<br>-37,14320609	36,5<br>29,3<br>32,0
Hybrid B3LYP With dispersion	Hydrog. Abstr.<br>Oxid. Addit.<br>2 O_2 molec.	-36,22804058	-0,49917117	-36,67134880<br>-36,68458647<br>-37,18178349	35,1<br>26,7<br>28,0

We see that within the GGA functionals set the activation energy barriers for the 3 mechanisms are only slightly affected by the functional-type: with respect to BP86 calculated activation barriers, PBE lowers the 3 values in the range from -1.2 to -3.0 kcal/mol, and BLYP slightly lowers the value for mechanism a) (-0.8 kcal/mol) and increases the values for mechanisms c) and c) + O_2 (+1.1 and +0.4 kcal/mol, respectively). The meta-GGA TPSS functional increases the energy barriers for mechanisms a) and c) + O_2 (+3.6 and +2.1 kcal/mol, respectively) and slightly decreases the barrier for mechanism c) (-0.3 kcal/mol). Both the hybrid

B3LYP and meta-hybrid M06 functionals significantly increase all the three energy barriers, with B3LYP in the range from +9.3 to +18.4 kcal/mol, and M06 in the range from +7.8 to +10.0 kcal/mol. Inclusion of D3-BJ dispersion on B3LYP calculations decreases all of the activation energy barriers of the same amount as that calculated for BP86 (from -1.4 to -4.0 kcal/mol).

Importantly, again the same activation energy barrier trend, namely $\Delta E^{\neq}$a) > $\Delta E^{\neq}$c) > $\Delta E^{\neq}$c) + O_2 is calculated with the PBE, BLYP, TPSS, and M06 functionals, whereas with B3LYP $\Delta E^{\neq}$c) + O_2 is slightly larger than $\Delta E^{\neq}$c) (2.7 kcal/mol and 1.3 kcal/mol including D3-BJ dispersion effects).

Solvation (benzene) effects.

We studied solvent effects on this reaction by performing SOC single point energy calculations with BP86 (without inclusion of D3-BJ dispersion) and with a TZ2P basis set on the DZP optimized reactants and transition state structures for the 3 mechanisms, using COSMO model (solvent = benzene, as in experiment). The results, reported in Table S4, where are compared to the gas-phase ones, show that inclusion of solvent effects decreases significantly the activation barrier only for the hydrogen abstraction mechanism by 4.9 kcal/mol, whereas the activation energy barriers for the oxidative addition mechanisms (with one and two dioxygen molecules) are only marginally affected (a decrease of 1.5 kcal/mol and an increase of 0.1 kcal/mol are calculated for the two paths, respectively).

Table S4: Solvent effects (COSMO model) on the energies of reactants and transition states and on the activation (electronic) energy barriers for the three studied mechanisms.

Level of theory	Mechanism	Energy LAuH (Hartree)	Energy O_2 (Hartree)	Energy TS (Hartree)	$\Delta E^{\neq}$ (kcal/mol)
BP86 without solvation	Hydrog. Abstr.<br>Oxid. Addit.<br>2 O_2 molec.	-3,60103601	-0,36207276	-3,919726667<br>-3,930258700<br>-4,303440488	27,2<br>20,6<br>13,6
BP86 with solvation	Hydrog. Abstr.<br>Oxid. Addit.<br>2 O_2 molec.	-3,60993783	-0,36201743	-3,936373148<br>-3,941561200<br>-4,312186870	22,3<br>19,1<br>13,7

Importantly, again the same activation energy barrier trend, namely $\Delta E^{\neq}$a) > $\Delta E^{\neq}$c) > $\Delta E^{\neq}$c) + O_2, is calculated also with inclusion of solvent (benzene) effects.
